# The Relationship Between the Functional Head Impulse Test (F-HIT) and Digital Gaming Addiction in Adolescents

**DOI:** 10.3390/children12070837

**Published:** 2025-06-25

**Authors:** Deniz Uğur Cengiz, Sanem Can Çolak, Mehmet Akif Kay, Büşra Kurtcu, Mehmet Sağlam, Munise Duran, Osman Tayyar Çelik

**Affiliations:** 1 Department of Audiology, Inonu University, 44000 Malatya, Türkiye; deniz.cengiz@inonu.edu.tr (D.U.C.); sanemcan.colak@inonu.edu.tr (S.C.Ç.); bkurtcu@gelisim.edu.tr (B.K.); 2 Department of Child Development, Batman University, 72000 Batman, Türkiye; mehmetakif.kay@batman.edu.tr; 3 Department of Child Development, Inonu University, 44000 Malatya, Türkiye; mehmet.saglam@inonu.edu.tr; 4 Department of Preschool, Inonu University, 44000 Malatya, Türkiye; munise.durdu@inonu.edu.tr

**Keywords:** f-HIT, vestibulo-ocular reflex, digital game, addiction, adolescent

## Abstract

**Background/Objectives**: Considering the extensive use of digital tools among adolescents and the effects of game addiction on physical, social, emotional, and cognitive domains, this study aimed to investigate the relationship between digital game addiction and the vestibulo-ocular reflex in high school students. **Methods**: In this descriptive relational study, the relationship between digital game addiction and the functional head impulse test was investigated in adolescents. Two groups of adolescents, with and without digital game addiction, were compared based on the functional head impulse test. The Digital Game Addiction Scale was administered to assess digital game addiction in adolescents aged 14 to 18 years. **Results**: The findings were analyzed statistically, and the results indicated a statistically significant relationship between digital game addiction and the vestibulo-ocular reflex, with digital game addiction negatively affecting the vestibulo-ocular reflex in adolescents. **Conclusions**: The findings indicate that digital game addiction in adolescents may impair VOR function, suggesting a potential negative impact on balance and perceptual processing. These results highlight the importance of early interventions and digital literacy programs to mitigate the adverse effects of excessive gaming during adolescence.

## 1. Introduction

Developments in technology have integrated it into virtually every area of life, leading to its widespread use by individuals across all levels of society and age groups. Children and youth are known to use digital tools more frequently than adults. With the rapid integration of technology into daily life, children born into this technological environment are often described by terms such as digital natives, Generation Z, or the gaming generation. Although the internet and digital tools facilitate daily life for both adults and children, they also pose various negative effects [1,2,3,4].

The intensive use of technology in children and adolescents from early ages has been reported to result in various adverse outcomes, including attention deficits and disorganization, delayed language skill acquisition, impaired development of creativity and imagination, negative effects on academic achievement, and an increased tendency toward aggressive and harmful behaviors [2,5,6,7,8]. While the excessive use of technology negatively affects the child’s time spent playing, eating, and sleeping and their cognitive and emotional development, it also damages cognitive functions such as impulse control, self-regulation, mental flexibility, and the ability to understand the thoughts and feelings of others. In addition, problems such as the shortening of attention spans, weakening of concentration, and increasing levels of distraction may occur [2,9,10,11]. The fact that especially attention span and perception processes are affected by the intense use of digital games highlights the severity of this effect. Prolonged exposure to digital screens necessitates continuous ocular accommodation and adaptation, progressively straining the visual–motor system. Sustained and intensive screen use may induce discrepancies within the visual–vestibular system, thereby impairing vestibulo-ocular reflex (VOR) function and compromising balance performance.

Balance is defined as the static and dynamic harmony between the human body and the environment, and it is achieved in three stages: obtaining information about the position/position change (informing), perceiving the information and preparing the balance center (interpretation), and giving appropriate motor responses to the interpreted information (application). Considering the influence of balance on the perception and interpretation of information, it is deemed important to control the factors that affect balance. The vestibulo-ocular reflex (VOR), assessed using the functional head impulse test (f-HIT), maintains coordination between head and eye movements, thereby enabling the perception of moving objects and contributing to postural stability [12,13,14]. The f-HIT device is a new test battery that functionally evaluates the VOR. VOR evaluation in different head accelerations simulating activities of daily living is possible with the f-HIT device. This device quantitatively measures the problem of individuals experiencing issues in dynamic and static visual acuity [15,16,17]. Due to these features of the f-HIT device, considering the further effects of heavily played digital games on adolescents and based on the fact that there is no study in the literature evaluating digital game addiction and vestibulo-ocular reflex, in this study, it was aimed to examine the relationship between digital game addiction and functional head impulse test in adolescents. In this context, the evaluation of VOR performance using the f-HIT device in individuals with digital game addiction addresses a significant gap in the field by enabling the objective assessment of visual–vestibular integration. The findings may enhance the understanding of the neurophysiological effects associated with digital game addiction.

## 2. Materials and Methods

### 2.1. Research Design

This research was designed as a descriptive relational study. The study examined the relationship between digital game addiction and functional head impulse test performance in adolescents. To this end, two groups of adolescents, those with and without digital game addiction, were compared based on their performance in the functional head impulse test.

### 2.2. Study Group

In the present study, the Digital Game Addiction Scale was administered to adolescents aged 14 to 18 years attending a high school in the Battalgazi District of Malatya Province, Türkiye. Based on the scale scores, a total of 76 male adolescents were included, comprising 38 adolescents with digital game addiction and 38 adolescents without digital game addiction. As all adolescents identified as addicted according to the scale were male, the control group was also composed exclusively of male adolescents. The inclusion of only male adolescents in this study was based on the significantly higher prevalence of digital game addiction among males and the difficulty in obtaining a sufficient sample of female participants. This approach was adopted to ensure sample homogeneity and enhance the statistical power of the data. Based on information obtained from psychological counselors in the school guidance service, individuals without cognitive or mental disorders, communication difficulties, or conditions affecting the auditory or vestibular systems were included in the study.

### 2.3. Data Collection Tools

#### 2.3.1. Digital Game Addiction Scale (DGAS)

The scale was developed by Lemmens et al. to assess problematic digital game playing behaviors in adolescents aged 12 to 18 years [18]. The Turkish adaptation was conducted by Irmak and Erdoğan (2015) [19]. The original scale comprises 21 items and seven sub-dimensions. In the present study, the 7-item short form of the DGAS-21 was utilized. The scale is a five-point Likert type with one-factor structure and it is scored between 1 and 5 (1 = never, 5 = always) (range: 7–35). Two formats, monothetic and polythetic, are used to diagnose whether an adolescent is a game addict based on the DGAS-7 scale. According to the monothetic diagnosis, if the person selects option 3 (sometimes) or more on seven of the seven items, and according to the polythetic diagnosis, if the person selects 3 (sometimes) and above in at least four of the seven items, they are defined as a game addict. In this study, 38 adolescents were determined as digital game addicts according to polythetical diagnosis.

#### 2.3.2. Functional Head Impulse Test (F-HIT)

A BEON Solutions srl (Zero Branco, Italy) brand f-HIT system was used. The computer containing this system software consisted of a gyroscope (headband sensor) and a mini keyboard. The gyroscope (headband sensor), used to measure the angular velocity and direction of head movements, was positioned at the center of the forehead. Participants were seated in a chair 1.5 m from the computer monitor. To assess static vision based on each participant’s viewing distance, visual acuity was determined by progressively reducing the size of the Landolt C character on the monitor until the smallest size the participant could clearly identify was reached. The Landolt C character appeared on the screen in 8 different ways as it shrank. The child was asked to determine the form of Landolt C characters by pressing the keys on the mini keyboard in their hand. After determining the static visual acuity of the child, their head was positioned according to lateral, posterior, and anterior semicircular canals (SSCs) and by making a head impulse at speeds of 4000–6000°/s^2^ for the lateral SSC and 3000–6000°/s^2^ for the posterior and anterior SSCs, and they were asked to select the form of Landolt C characters they saw on the screen by clicking on relevant buttons on the mini keyboard. The Landolt C character was displayed for 80 ms as the child’s head was moving. In the evaluation of this test, the percentage of correct answers (%CA) was calculated according to both the visual ratings of each stimulated frequency and the mean values of all frequencies in the stimulated channel. Prior to the assessment, practical trials were conducted to ensure that participants comprehended and accurately performed the test procedures.

### 2.4. Ethical Principles of Research

Ethics committee approval was obtained from the İnönü University Health Sciences Non-Interventional Clinical Research Ethics Committee with the decision number 2022/3461 dated 12 April 2022. A consent form was obtained from the families of the participants who agreed to participate in the study after obtaining permission from the Battalgazi District Governorship.

### 2.5. Data Analysis

Data analysis was conducted using the Statistical Program for Social Sciences (SPSS) version 25. The Kolmogorov–Smirnov test was applied to assess the normality of the data distribution [20]. The significance level (*p*) for comparison tests was set at 0.05. As the variables did not exhibit a normal distribution (*p* > 0.05), subsequent analyses were carried out using non-parametric test methods. Comparisons between independent groups were performed using the Mann–Whitney U test due to the violation of the normality assumption. For the analysis of categorical data, cross-tabulations were created, and chi-square (χ^2^) analysis was conducted.

## 3. Results

The participants were divided into two main groups: game addicts (*n* = 38) and non-game addicts (*n* = 38). The comparison of DGAS scores and age groups is presented in Table 1. A statistically significant difference was observed between the groups in DGAS scores (*p* < 0.05). No statistically significant difference was found between the groups regarding age (*p* > 0.05), indicating a homogeneous distribution between the groups.

The comparison of groups based on participants’ play time, gaming device, and academic achievement is presented in Table 2. The participants demonstrated a homogeneous distribution in terms of gaming device type and age. It was found that 84.2% played digital games seven days a week, while 15.8% played three to five days a week. The rate of those who played games for 4–6 h a day was 63.2%, whereas the rate of those who played games for 1–3 h was 15.8%. It was found that 47.4% of those who were not game addicts played digital games 7 days a week and 15.8% played digital games 3–5 days a week. It was observed that the rate of those who played 1–3 h a day was 57.9%, and the rate of those who played 4–6 h and less than 1 h was 21.1%. Statistically significant differences were observed between the groups regarding weekly playing time, daily playing time, grade point average, and playing duration in years (*p* < 0.05). No statistically significant difference was found between the groups concerning the gaming device used (*p* > 0.05).

The comparison of the measurements of the lateral, posterior, and anterior SSCs at different speeds in the f-HIT is shown in Table 3. Table 3 also presents the effect size of digital addiction on the percentage of correct responses in the f-HIT test. The calculated d value is interpreted as follows: 0.20 indicates a small effect size, 0.50 a medium effect size, and 0.80 a large effect size [21]. Statistically significant differences were identified between the groups in lateral SSC 4000°/s^2^ and total %CA values, posterior SSC 3000°/s^2^ and 6000°/s^2^ %CA values, and anterior SSC 5000°/s^2^ %CA values (*p* < 0.05; Table 3). No statistically significant differences were observed between the groups for lateral SSC 5000°/s^2^, 6000°/s^2^, posterior SSC 4000°/s^2^, 5000°/s^2^, and total %CA values, as well as anterior SSC 3000°/s^2^, 4000°/s^2^, 6000°/s^2^, and total %CA values (*p* > 0.05).

**Figure 1:**
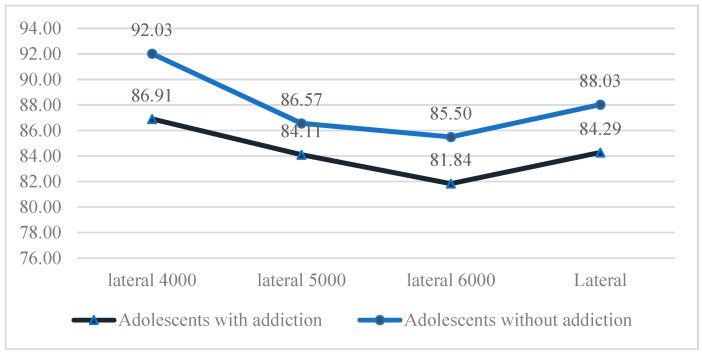
Comparison of groups in terms of lateral SSC percentage correct answers.

Total %CA values related to lateral SSC 4000°/s^2^, 5000°/s^2^, and 6000°/s^2^ were lower in individuals with digital game addiction compared to the control group. A statistically significant difference was identified between the groups for lateral SSC 4000°/s^2^ and total %CA values (*p* < 0.05). No statistically significant differences were observed between the groups for lateral SSC 5000°/s^2^ and 6000°/s^2^ %CA values (*p* > 0.05; see Figure 1).

**Figure 2:**
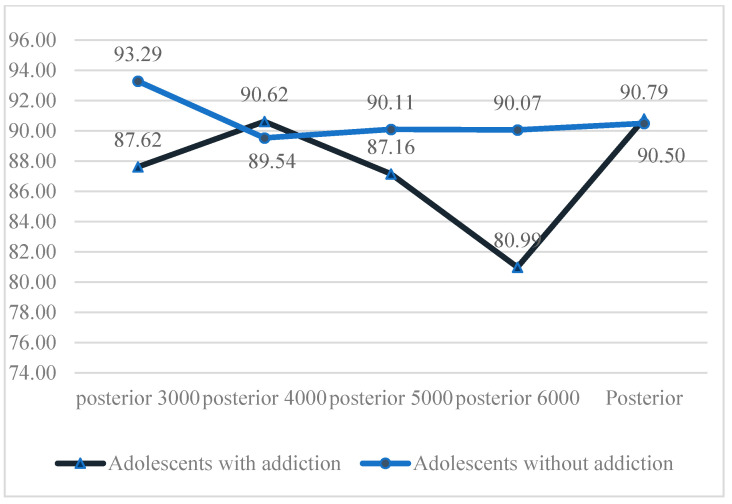
Intergroup comparison of posterior SSC percentage of correct answers.

Total %CA values for posterior SSC 3000°/s^2^, 5000°/s^2^, and 6000°/s^2^ were lower in individuals with digital game addiction compared to the control group. Statistically significant differences were identified between the groups for posterior SSC 3000°/s^2^ and 6000°/s^2^ %CA values (*p* < 0.05). No statistically significant differences were observed between the groups for posterior SSC 4000°/s^2^ and 5000°/s^2^ total %CA values (*p* > 0.05; see Figure 2).

**Figure 3:**
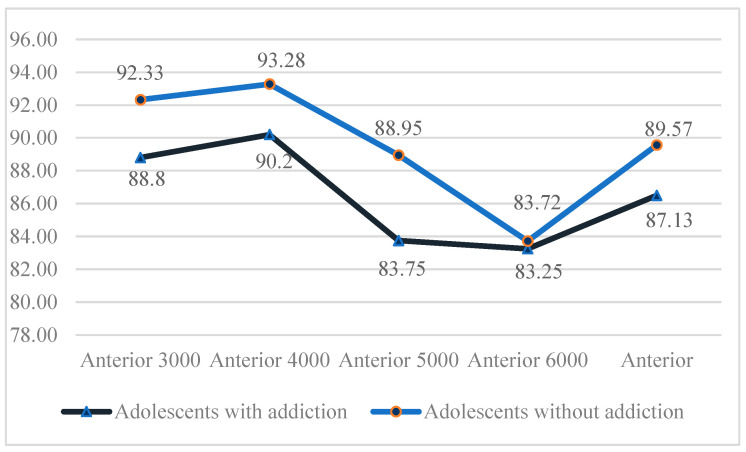
Intergroup comparison of anterior SSC percentage of correct answers.

Total %CA values for anterior SSC 3000°/s^2^, 4000°/s^2^, 5000°/s^2^, and 6000°/s^2^ were lower in individuals with digital game addiction compared to the control group. A statistically significant difference was observed between the groups for the anterior SSC 5000°/s^2^ value (*p* < 0.05). No statistically significant differences were found between the groups for anterior SSC 3000°/s^2^, 4000°/s^2^, and 6000°/s^2^ total %CA values (*p* > 0.05; see Figure 3).

## 4. Discussion

Children are born with psychomotor abilities such as attention, coordination, and flexibility, which develop further through play. Play facilitates the development of balance and coordination, enhancing motor skills and agility, while also enabling children to explore and understand their environment [22]. However, environmental changes and digitalization have altered play patterns, leading to the increasing prevalence of digital games among children.

Although DGAS scores differed significantly between groups, no significant differences were observed in age or type of gaming device, indicating a homogeneous group distribution consistent with the literature. Technological access varies by gender; boys, who encounter less societal control and have easier access to technology, tend to engage more frequently in virtual environments [1]. Studies examining different age groups in the literature similarly demonstrate that boys exhibit higher levels of digital game addiction compared to girls [23,24,25].

Statistically significant differences were found between the groups in terms of weekly and daily playing time, grade point average, and years of digital game use. The designation of some individuals as addicts reflects their significantly longer engagement with digital games. Similarly, data from the Turkish Statistical Institute (2021) show that mobile phone/smartphone usage increases with age: 53.9% in children aged 6–10, 75.0% in those aged 11–15, and 64.4% overall for ages 6–15. Among users, 84.6% use their devices daily [26]. A study with secondary school students found that children spending 3 h or more daily on digital devices were more likely to be addicted than those using them for less than 1 h, especially when the device was used for play rather than studying [27]. The We Are Social 2022 report indicates growing global digital engagement: 67.1% of the world owns a mobile phone, social media users increased by over 10% in one year, and the average user spends 4 h and 48 min daily on mobile devices [28].

Findings indicated that smartphones were the most frequently used device in both the addicted (76.3%) and non-addicted (89.5%) groups. Computers ranked second in the addicted group (13.2%), while tablet use was comparable between the groups. Gökel (2020) reported that children’s primary digital activities involved the internet, smartphones, tablets, and game consoles [29]. The We Are Social 2022 report also emphasized the global rise in mobile device usage [28]. Erkisi and Sağlam (2020) found significantly higher loss of control and internet addiction scores among smartphone users compared to non-users [30]. These findings highlight the widespread use of digital tools, especially among children and adolescents, aligning with the current study results.

Another finding of the study was that children with higher digital game addiction scores exhibited lower academic achievement. Similar studies have reported an inverse relationship between digital addiction and academic performance, with problematic use contributing to difficulties in academic and professional life [31,32,33,34]. The lower average academic success observed in the addicted group supports this association. Addiction has been noted to interfere with homework completion, diminish classroom attention, and impair academic perception [35].

The fact that adolescents who play games at the level of addiction do not have any complaints subjectively does not objectively indicate that they are healthy because gaze stabilization does not indicate clear vision. In some cases, even if the VOR gain is normal, blurred vision may occur with head movement. In order to quantitatively reveal this situation, the f-HIT device, which is a perceptual test that functionally evaluates the VOR at different speeds and accelerations, was developed [16]. In the study, lateral SSC 4000°/s^2^ %CA, lateral SSC total %CA, posterior SSC 3000°/s^2^ %CA, posterior SSC 6000°/s^2^ %CA, and anterior SSC 5000°/s^2^ %CA of adolescents addicted to digital games were statistically lower compared to non-addicted peers. An examination of the other acceleration values reveals that individuals with digital game addiction exhibit lower scores; however, these differences do not reach statistical significance. This outcome is considered to be related to the sensitivity of the measurement. Functionally adverse effects are observed in different acceleration values of vestibulo-ocular reflexes of children addicted to digital games. In other words, it was determined that digital game addiction negatively affected the vestibulo-ocular reflex in adolescents. In the study conducted by Gedik Toker and colleagues, it was reported that, although no significant changes were observed in VOR gain values when daily digital screen use exceeded six hours, a significant decrease in dynamic visual acuity scores was identified [36]. The findings of the present study are consistent with these results in the literature. Digital game addiction may impair vestibulo-ocular reflex (VOR) function and balance by inducing inconsistencies in the visual–vestibular system as a result of prolonged and intensive screen exposure. This effect is particularly evident in the functionality of the VOR. Another study reported that prolonged daily smartphone use leads to deterioration in postural control and significant alterations in balance parameters [37]. These findings indicate that increased screen time and reduced physical activity associated with digital game addiction disrupt the balanced functioning of visual–vestibular integration, thereby adversely affecting VOR function and balance. Hand–eye coordination, visual memory, and perception are of great importance for the application of the f-HIT device and for obtaining correct results. Mustafaoğlu and Yasacı (2018) concluded that playing digital games may cause physical health problems such as musculoskeletal system problems in body parts such as the spine, shoulder, hand, and wrist, dryness, pain, and redness in the eyes, and deterioration in sleep quality [38]. Constant exposure to screens has been shown to have adverse health effects, including the occurrence of epileptic seizures, concentration difficulties, and long-term mental health problems [39]. Additionally, in the study conducted by Toran et al., some mothers reported that digital games contributed to children’s conceptual development, hand–eye coordination, and visual memory [40]. In other studies, similar results have been obtained, and it is seen that digital games played in a controlled way increase hand–eye coordination and have many positive aspects that improve spatial skills [41]. It is noteworthy that playing digital games not at the level of addiction can also have positive effects. One of the most important indicators determining the direction of these effects is seen as “addiction”. Evaluating addicted adolescents with an objective vestibular test battery in the present study provided quantitative information. In addition, it should not be ignored that the perceptual process plays an important role in the application of the f-HIT device. In the light of the findings obtained in the study, it is seen that digital game addiction negatively affects the balance and perceptual process.

## 5. Conclusions

Changes in environmental conditions directly influence the developmental process in children. Technological advancements and the ongoing digital revolution introduce new environmental factors, progressively increasing their impact on children. Adolescence is characterized by a rapid developmental trajectory and changes across multiple domains, during which susceptibility to digital game addiction may increase, potentially leading to neurological effects. In the present study, digital game addiction was found to exert a negative impact on vestibulo-ocular reflex function. The impact on the vestibulo-ocular reflex, which plays a critical role in maintaining balance, indicates that digital game addiction may contribute to balance disorders. To mitigate such issues, it is essential to implement digital literacy education for both children and parents from an early age. Given the limited self-control typically observed during adolescence, monitoring and regulating adolescents’ interactions with technology would be beneficial. Considering that the need for play is a fundamental requirement for children, opportunities can be created to fulfill this need through peer interactions and non-digital games. Additionally, adolescents can be encouraged to engage in activities involving sports and physical exercise to support the development of balance.

## Figures and Tables

**Table 1:** Comparison of digital game addiction scale scores according to age.

Variable	Game Addict		Non-Game Addict		Test Value	*p* Value
	Mean ± SD M (Min–Max)	Cronbach α	Mean ± SD M (Min–Max)	Cronbach α		
Digital Game Addiction Scale (DGAS) Score	26.34 ± 5.16 26 (17–35)	0.785	12.16 ± 3.99 11.5 (7–21)	0.714	16.500	0.001 *
Age	Mean ± SD M (Min–Max)		Mean ± SD M (Min–Max)		Test Value	*p* Value
	15.63 ± 1.05 16 (14–17)		15,5 ± 1.16 15.5 (14–17)		675,000	0.610

SD: standard deviation, M: median, Min; *p* value: Mann–Whitney U test significance value, * *p* < 0.05: statistically significant difference between groups.

**Table 2:** Comparison of groups regarding information about participants’ digital play times, gaming device, and academic achievement.

Variable		Game Addict	Non-Game Addict	Total	χ^2^ Test	*p* ^a^ Value
		Number (%)				
Weekly Game Play Time (Days)	1 day	(-)	4 (%10.5)	4 (%5.3)	0.486	0.001 *
	1–3 days	(-)	10 (%26.3)	10 (%13.2)		
	3–5 days	6 (%15.8)	6 (%15.8)	12 (%15.8)		
	7 days	32 (%84.2)	18 (%47.4)	50 (%65.8)		
Daily Game Play Time (Hours)	Less than 1 h	(-)	8 (%21.1)	8 (%10.5)	0.660	0.001 *
	1–3 h	6 (%15.8)	22 (%57.9)	28 (%36.8)		
	4–6 h	24 (%63.2)	8 (%21.1)	32 (%42.1)		
	More than 7 h	8 (%21.1)	(-)	8 (%10.5)		
Gaming Device	Smartphone	29 (%76.3)	34 (%89.5)	63 (%82.9)	0.205	0.201
	Tablet	4 (%10.5)	3 (%7.9)	7 (%9.2)		
	Computer	5 (%13.2)	1 (%2.6)	6 (%7.9)		
Variable	Game Addict		Non-Game Addict		Test Value	*p* ^b^ Value
	Mean ± SD M (Min–Max)					
Average GPA	55.39 ± 9.36 55.5 (39.38–74.11)		66.36 ± 12.41 61.5 (51–91.7)		375,000	0.001 *
Game Play Time (Years)	5.66 ± 2.13 5 (3–13)		2.47 ± 1.13 3 (1–5)		90,500	0.001 *

*p* ^a^: chi-square test value (χ^2^), SD: standard deviation, *p* ^b^: Mann–Whitney U test, *p* value, * *p* < 0.05: a statistically significant difference between the groups.

**Table 3:** Comparison of the groups in terms of the f-HIT test percentage correct answer.

Variable	Game Addict	Non-Game Addict	Test Value	*p* Value	Cohen’s d
	Mean ± SD M (Min–Max)	Mean ± SD M (Min–Max)			
Lateral SSC 4000°/s^2^ %CA	86.91 ± 11.96 80 (60–100)	92.03 ± 12.71 100 (50–100)	2240.000	0.008 *	0.41
Lateral SSC 5000°/s^2^ %CA	84.11 ± 11.62 82.5 (50–100)	86.57 ± 14.1 88 (50–100)	2495.000	0.138	0.19
Lateral SSC 6000°/s^2^ %CA	81.84 ± 15.72 80 (50–100)	85.5 ± 15.73 84 (50–100)	2603.000	0.263	0.23
Lateral SSC Total %CA	84.29 ± 8.97 83.33 (60–100)	88.03 ± 9.6 90 (61.67–100)	2180.500	0.009 *	0.40
Posterior SSC 3000°/s^2^ %CA	87.62 ± 16.49 100 (50–100)	93.29 ± 13.01 100 (50–100)	2398.000	0.027 *	0.38
Posterior SSC 4000°/s^2^ %CA	90.62 ± 12.27 100 (57–100)	89.54 ± 16.09 100 (10–100)	2885.000	0.990	0.07
Posterior 5000°/s^2^ %CA	87.16 ± 16.14 100 (50–100)	90.11 ± 12.39 100 (67–100)	2687.000	0.415	0.20
Posterior SSC 6000°/s^2^ %CA	80.99 ± 17.82 75 (33–100)	90.07 ± 15.29 100 (50–100)	2109.000	0.001 *	0.54
Posterior SSC Total %CA	90.79 ± 7.5 91.75 (71.4–100)	90.5 ± 7.77 91.85 (72.2–100)	2820.500	0.802	0.03
Anterior SSC 3000°/s^2^ %CA	88.8 ± 15.19 100 (50–100)	92.33 ± 13.64 100 (50–100)	2569.000	0.153	0.24
Anterior SSC 4000°/s^2^ %CA	90.2 ± 13.09 100 (50–100)	93.28 ± 11.56 100 (67–100)	2537.000	0.126	0.24
Anterior SSC 5000°/s^2^ %CA	83.75 ± 16.66 83 (33–100)	88.95 ± 14.31 100 (50–100)	2367.500	0.039 *	0.33
Anterior SSC 6000°/s^2^ %CA	83.25 ± 19.42 100 (33–100)	83.72 ± 18.64 100 (50–100)	2879.000	0.971	0.02
Anterior SSC Total %CA	86.5 ± 10.97 87.13 (67–100)	89.57 ± 8.93 91.75 (72.25–100)	2449.500	0.103	0.30

SD: Standard Deviation, M: Median, *p* Value: Mann–Whitney U test significance value, * *p* < 0.05: a statistically significant difference between the groups.

## Data Availability

The raw data supporting the conclusions of this article will be made available by the authors on request.

## Abbreviations

The following abbreviations are used in this manuscript:

%CA	Percentage of Correct Answers
DGAS	Digital Game Addiction Scale
f-HIT	Functional Head Impulse Test
SPSS	Statistical Program for Social Sciences
VOR	Vestibulo-ocular Reflex
